# Ventricular arrhythmogenesis in pulmonary hypertension: From right ventricular remodeling to clinical implications

**DOI:** 10.14814/phy2.71067

**Published:** 2026-08-19

**Authors:** Abdulkadir Kiris, Peng Zhang, Peter Bronk, Bum‐Rak Choi, Gaurav Choudhary

**Affiliations:** ^1^ Research Service VA Providence Medical Center Providence Rhode Island USA; ^2^ Cardiovascular Research Center Brown University Health Cardiovascular Institute Providence Rhode Island USA; ^3^ Division of Cardiology, Department of Medicine Warren Alpert Medical School of Brown University Providence Rhode Island USA

**Keywords:** pulmonary hypertension, right ventricle, ventricular arrhythmia

## Abstract

Pulmonary hypertension (PH) results in chronic pressure overload of the right ventricle, leading to progressive structural, metabolic, neurohumoral, and electrophysiological remodeling. Although right ventricular failure is the major determinant of outcomes in PH, these remodeling processes may also create a substrate for ventricular arrhythmogenesis, thereby contributing to poor outcomes. While atrial arrhythmias are more common, ventricular arrhythmias (VAs) have also been reported in PH and may have important clinical implications. However, their epidemiology, mechanisms, and optimal management remain incompletely understood. This review summarizes pathophysiological mechanisms of VAs in PH by integrating findings from preclinical and clinical investigations. Available data show that premature ventricular contractions and non‐sustained ventricular tachycardia are the most frequently reported VAs, whereas sustained ventricular tachycardia and ventricular fibrillation appear to be uncommon. Arrhythmia susceptibility in PH is associated with several mechanisms that provide possible therapeutic targets. These include changes in specific ion channels in the right ventricle, abnormalities in calcium handling, structural remodeling characterized by hypertrophy, fibrosis and gap junction alterations, autonomic nervous system imbalance, myocardial ischemia, inflammation, and oxidative stress. We discuss the translational implications of these findings and highlight gaps in knowledge to guide future research on the epidemiology, targeted management, and prognostic significance of VAs in PH.

## 1 INTRODUCTION

Pulmonary hypertension (PH) is defined by a resting mean pulmonary artery pressure (mPAP) >20 mmHg as measured by right heart catheterization (Humbert et al., 2022). PH affects approximately 1% of the global population, with prevalence increasing markedly with age (Humbert et al., 2022; Simonneau et al., 2019). PH is classified into five distinct groups based on its underlying etiology: pulmonary arterial hypertension (PAH), PH due to left heart disease, PH associated with lung disease or hypoxia, chronic thromboembolic PH, and PH with multifactorial and/or unclear mechanisms (Leber et al., 2021). Despite advances in management, PH remains associated with considerable morbidity, mortality, and healthcare burden. In the absence of treatment, the median survival following diagnosis is approximately 3 years, with five‐year survival rates ranging from 19% to 59%, depending on disease subtype and severity (Gall et al., 2017; Lau et al., 2017).

Chronic right ventricular (RV) pressure overload resulting from PH initially leads to adaptive RV hypertrophy and eventually results in maladaptive remodeling characterized by chamber dilation and contractile dysfunction (Humbert et al., 2022). Ultimately, right heart failure represents the major determinant of morbidity and mortality in patients with PH. Beyond mechanical RV dysfunction, RV remodeling is accompanied by profound electrophysiological alterations, including ion‐channel remodeling, myocardial fibrosis, inflammation, impaired myocardial perfusion, and autonomic dysregulation, which collectively create a proarrhythmic substrate.

In addition to progressive RV failure, cardiac arrhythmias are increasingly recognized as important complications across the spectrum of PH (Andersen et al., 2021; Bandorski et al., 2023; Bandorski, Bogossian, Ecke, et al., 2016; Bandorski, Bogossian, Stempfl, et al., 2016; Baskar et al., 2017; Cannillo et al., 2015; Chaturvedi et al., 2020; Chen et al., 2022; Chiriac et al., 2020; Fingrova et al., 2021; Havranek et al., 2020; James, 1962; Kanemoto & Sasamoto, 1979; Kanmanthareddy et al., 2014; Mercurio et al., 2018; Naranjo et al., 2022; Olsson et al., 2013; Rottlaender et al., 2012; Ruiz‐Cano et al., 2011; Sammut et al., 2023; Smith et al., 2018; Tongers et al., 2007; Wen et al., 2014; Xue et al., 2022; Yi et al., 2014). Although atrial arrhythmias remain the most frequently encountered rhythm disturbances, ventricular arrhythmias (VAs) have received comparatively less attention despite accumulating evidence supporting their clinical relevance (Abouzaid et al., 2024; Cirulis et al., 2019; Kiris et al., 2026; Rajdev et al., 2012; Wanamaker et al., 2018). The reported spectrum of VAs ranges from premature ventricular complexes (PVCs) and repolarization abnormalities, including QT interval prolongation, to non‐sustained and sustained ventricular tachycardia (VT) and ventricular fibrillation (VF). Rather than representing isolated electrical events, these arrhythmias may reflect the clinical manifestation of an underlying arrhythmogenic substrate resulting from pressure overload‐induced RV remodeling. Accordingly, VAs may represent not only markers of advanced RV remodeling but also potential contributors to clinical deterioration.

In the general population, isolated PVCs are usually considered benign in the absence of structural heart disease. In contrast, VAs occurring in structurally remodeled ventricles are associated with adverse cardiovascular outcomes (Bikkina et al., 1993). A similar paradigm is likely applicable to PH, where chronic pressure overload‐induced RV remodeling may predispose patients to ventricular arrhythmogenesis and potentially contribute to hemodynamic deterioration and sudden cardiac death, leading to worse long‐term outcomes. Nevertheless, the epidemiology, mechanisms, prognostic significance, and optimal management of VAs in PH remain incompletely understood. Current evidence is largely derived from small, heterogeneous observational studies, precluding definitive conclusions regarding their clinical significance and optimal management.

In this review, we provide a comprehensive translational overview of VAs in PH by integrating evidence from basic, translational, and clinical studies. We summarize the current evidence regarding their epidemiology and clinical impact, discuss the structural, electrophysiological, and molecular mechanisms underlying ventricular arrhythmogenesis, review current approaches to evaluation and management, and highlight existing knowledge gaps and future research priorities aimed at improving risk stratification and therapeutic strategies in this high‐risk population.

## 2 EPIDEMIOLOGY

Available epidemiological data regarding VAs in PH populations are summarized in Table 1 (Andersen et al., 2021; Bandorski et al., 2015; Bandorski et al., 2023; Bandorski, Bogossian, Stempfl, et al., 2016; Folino et al., 2003; Umar et al., 2012; Xue et al., 2022; Yi et al., 2014). Among the reported VAs in PH, PVCs are the most frequently reported subtype, with a prevalence of up to 78.5%, followed by non‐sustained VT (NSVT). In contrast, data on sustained VT and VF are limited, suggesting that these malignant arrhythmias appear to be relatively uncommon in PH patients. In a multicenter survey of patients with PAH, VF accounted for only 8% of cardiopulmonary arrest cases requiring cardiopulmonary resuscitation (Hoeper et al., 2002). Similarly, a recent report demonstrated that VT or VF was the initial rhythm in only 1 out of 26 patients (4%) with PAH undergoing cardiopulmonary resuscitation (Yang et al., 2022). Notably, both studies included only PAH patients, and the lack of epidemiological data in other PH subgroups precludes meaningful comparisons across different PH etiologies. In contrast, in the general population, VF and VT account for approximately 45% and 0.6% of initial arrest rhythms, respectively, based on a prospective, randomized multicenter study (Vukmir, 2009). In patients with left ventricular heart failure, VF or VT is reported as the initial rhythm in up to 37.5% of cases (Woolcott et al., 2020).

**TABLE 1:** Prevalence (%) of ventricular arrhythmias.

	Study design	PH groups studied	PVC	NSVT	VT	VF
Folino et al. (2003)	Prospective, observational case–control	1	44.4			
Bandorski et al. (2015)	Prospective, observational	1,3,4 and 5	45.6	13		
Bandorski, Bogossian, Stempfl, et al. (2016)	Retropective, observational	1 and 4		15.4		
Andersen et al. (2021)	Prospective, observational	1and 4		8.8		
Xue et al. (2022)	Prospective, observational	1		2.3		0.14
		2		16.7		
		3		3.1		
		4		1.9		
Bandorski et al. (2023)	Retropective, observational	1 and 4	78.5			

*Note*: PH groups: 1, pulmonary arterial hypertension; 2, PH due to left heart disease; 3, PH associated with lung disease or hypoxia; 4, chronic thromboembolic PH; 5, PH with multifactorial and/or unclear mechanisms. In the study by Folino et al., the reported prevalence refers to the proportions of patients with >700 PVCs per 24 h. In the study by Bandorski et al. (2015), 45.6% represents the proportion of patients with detected PVCs, and the mean PVC count among these patients was 70 beats during 72‐h Holter monitoring.

Abbreviations: NSVT, non‐sustained ventricular tachycardia; PH, pulmonary hypertension; PVC, premature ventricular contraction; VF, ventricular fibrillation; VT, sustained ventricular tachycardia.

Current epidemiological data also suggest that the prevalence and incidence of PAH are increasing disproportionately among older adults, which is associated with higher morbidity and mortality (Yu et al., 2025; Zhou et al., 2025). However, the contribution of VAs to this increased risk in elderly PAH patients remains unclear. Observational data indicate that PH patients who develop VAs tend to be older than those who do not, suggesting that aging may be a potential risk factor for VA development in PH, although this association has not consistently reached statistical significance (Andersen et al., 2021; Bandorski et al., 2015; Bandorski, Bogossian, Stempfl, et al., 2016). Although clinical epidemiological data remain limited, these observations provide a rationale for investigating how RV remodeling in PH may promote ventricular arrhythmogenesis.

## 3 MECHANISMS OF VENTRICULAR ARRHYTHMOGENESIS IN PULMONARY HYPERTENSION

**FIGURE 1:**
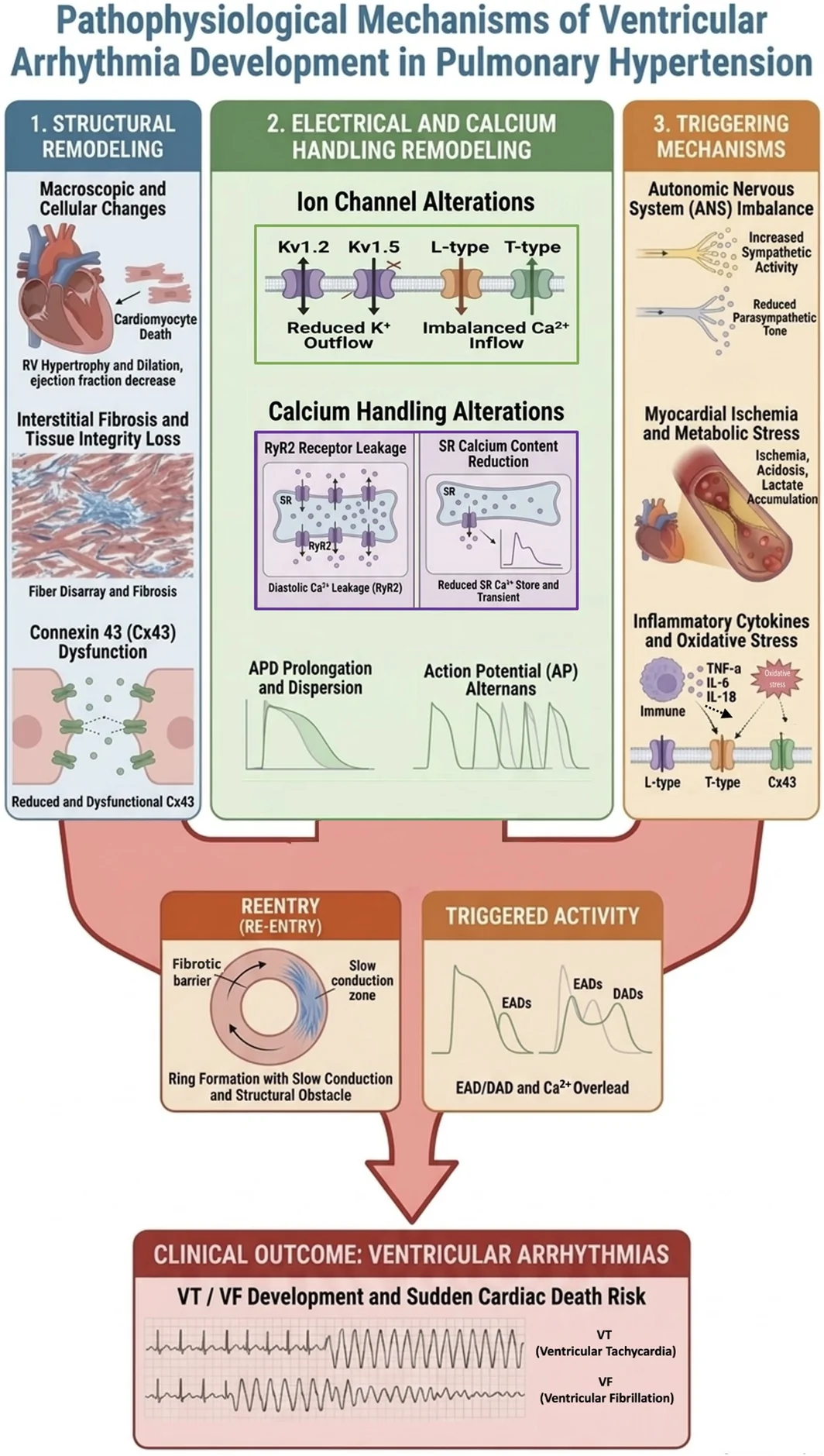
Development of ventricular arrhythmogenic substrate in pulmonary hypertension. Pulmonary hypertension (PH)‐induced right ventricular remodeling is characterized by structural changes (hypertrophy, interstitial fibrosis, and connexin dysfunction), alterations in electrical activity and calcium handling (action potential prolongation and alternans resulting from ion channel remodeling, RyR2 leak, and reduced sarcoplasmic reticulum calcium content), and multiple arrhythmia‐triggering mechanisms, including autonomic nervous system imbalance, myocardial ischemia, inflammation, oxidative stress, and metabolic stress. Collectively, these alterations promote reentrant circuits and triggered activity, ultimately leading to ventricular tachycardia, ventricular fibrillation, and an increased risk of sudden cardiac death.

In PH, the RV undergoes complex electrical, mechanical, and structural remodeling accompanied by systemic neurohormonal activation and autonomic nervous system (ANS) dysregulation. These alterations collectively create an arrhythmogenic substrate, thereby contributing to the increased risk of VAs observed in this population (Figure 1).

### 3.1 Development of arrhythmogenic substrate

#### 3.1.1 Structural remodeling

In response to sustained pressure overload in PH, the RV undergoes progressive structural remodeling. At the organ level, this is manifested by RV hypertrophy and dilatation. These changes are underpinned by a spectrum of cellular and tissue‐level alterations, including cardiomyocyte hypertrophy, apoptosis, necrosis, interstitial fibrosis, capillary rarefaction, and myocardial fiber disarray (Akazawa et al., 2020; Benoist et al., 2011; Benoist et al., 2012; Graham et al., 2018; Houston et al., 2023; Luesebrink et al., 2012; Qin et al., 2022; Sasano et al., 2007; Strauss et al., 2022; Tanaka et al., 2013; Tanaka et al., 2022; Umar et al., 2012; Wu et al., 2022).

In addition to these cellular and extracellular changes, PH also damages intercellular connections by gap junction remodeling. Connexin 43 (Cx43), the predominant gap junction protein in ventricular myocytes, plays a pivotal role in cell‐to‐cell electrical coupling. Qualitative and quantitative alterations in Cx43 have been documented in experimental models of PH. In the monocrotaline (MCT) rat model, Sasano et al. reported that dephosphorylation of Cx43 resulted in its inactive form and internalization into the cytoplasm of RV cardiomyocytes (Sasano et al., 2007). Similarly, in the sugen/pneumonectomy (Su/Pn) model, Strauss et al. observed a significant reduction in the phosphorylated (active) fraction of Cx43, while its total expression was not altered (Strauss et al., 2022). Recently, Wu et al. demonstrated degradation and disorganization of Cx43 in the MCT model, further supporting gap junction disruption in impaired electrical coupling (Wu et al., 2022).

Myocyte death, fibrosis, and hypertrophy along with alterations in connexins disrupt myocardial electrical architecture by reducing gap‐junction coupling and increasing anisotropy, promoting heterogeneous conduction. These changes are characterized by regional differences in action potential duration (APD), refractory period (RP), and conduction velocity (CV), which results in slow conduction and localized conduction block thereby creating a substrate for reentry (Remo et al., 2012; Zhang et al., 2023). Umar et al. demonstrated that spontaneous VF in the MCT model is sustained by a combination of focal activity and incomplete reentrant circuits (Umar et al., 2012). On the other hand, Strauss et al. reported that once initiated, VT/VF episodes in the Su/Pn model are maintained by stable reentrant circuits (Strauss et al., 2022). Therefore, RV structural remodeling likely plays an important role in the maintenance of VAs in PH.

#### 3.1.2 Electrophysiological remodeling

##### Ion channel alterations

RV cardiomyocytes possess distinctive electrophysiological characteristics compared with the left ventricle (LV). RV cardiomyocytes have a higher expression of certain potassium channels involved in action potential (AP) generation such as Kv4.2 and Kv4.3 (fast I_to_ channels) and KCNQ1 (a part of I_Ks_ complex) compared to left ventricle (Molina et al., 2016). In addition, there are differences in the regulation of cardiomyocyte Ca^2+^ handling due to distinct rates of cyclic adenosine monophosphate degradation in RV and LV (Molina et al., 2014). Electrical remodeling of the RV in PH is characterized by APD prolongation, increased APD dispersion, and the emergence of AP alternans, all of which are associated with increased susceptibility to VAs (Benoist et al., 2011; Benoist et al., 2012; Strauss et al., 2022; Umar et al., 2012).

APD prolongation arises from alterations in potassium and calcium ion channel currents in the RV in settings of PH (Benoist et al., 2011; Lee et al., 1997; Lee et al., 1999; Zhang et al., 2004). It has been shown that messenger ribonucleic acid and protein expression of key potassium channels such as Kv1.2, Kv1.5, Kv2.1, Kv4.2, Kv4.3, and Kv1.4 is decreased in the RV from MCT‐ and Su/Pn‐induced PH rat models (Benoist et al., 2011; Lee et al., 1997; Lee et al., 1999; Strauss et al., 2022; Umar et al., 2012). Also, calcium channel expression is disrupted, with decreased expression of L‐type and increased expression of T‐type voltage‐gated Ca^2+^ channels in the RV from the MCT model (Benoist et al., 2011). Recent studies by Park et al. have further demonstrated decreased expression of genes encoding Ca^2+^ channels, as well as differential regulation of K^+^ channel subtypes in both animal models (MCT and Sugen/hypoxia) and in patients with PAH (Park et al., 2023).

The ion channel alterations also occur in a temporally and spatially heterogeneous manner. Lee et al. reported that during early progression of PH (around 14 days in the MCT model) APD prolongation is primarily driven by alterations in Ca^2+^ channel. In contrast, reductions in potassium channel (Kv1.4) expression become dominant in later stages (around 28 days) (Lee et al., 1997). Therefore, APD prolongation in the early stage of PH‐induced RV hypertrophy is mainly due to increased Ca^2+^channel density, whereas decreased Kv1.4 density plays a more significant role in the late stage, suggesting different mechanistic targets based on the disease stage.

In addition to prolongation of APD, there are regional differences in expression of potassium channels in the RV from PH animals. Reduced expression of Kv4.2 and Kv4.3 was observed specifically in the RV free wall, but not in the septum or LV. These differences likely result in increased APD dispersion within the RV and between ventricles (Benoist et al., 2011; Strauss et al., 2022; Zhang et al., 2004) and may facilitate reentry, resulting in ventricular arrhythmias.

Prolonged APD and increased APD dispersion are reflected by QT interval abnormalities on surface electrocardiography and are associated with an increased risk of VAs (Varro et al., 2021). Consistent with this, corrected QT (QTc) and QTc dispersion (QTcd) are significantly prolonged in patients with PH, suggesting impaired ventricular repolarization (Hong‐liang et al., 2009; Yi et al., 2014). Moreover, both parameters correlate positively with mPAP in women, but not in men (Hong‐liang et al., 2009). Although a direct association between QTc abnormalities and the incidence of VAs in PH has not yet been definitively established, prolonged QTc and QTcd may reflect underlying ion channel alterations and electrical remodeling, leading to abnormalities in ventricular repolarization, thereby indicating the presence of a substrate that predisposes patients to malignant arrhythmias and adverse clinical outcomes. Supporting the clinical relevance of these findings, progressive QTc prolongation during follow‐up has been identified as an independent predictor of mortality in patients with PAH (Hinojosa et al., 2025). Collectively, these findings suggest that QTc abnormalities provide indirect clinical evidence of electrical remodeling in PH.

AP alternans, involving beat‐to‐beat variations in AP duration and amplitude, further contributes to electrical instability. Discordant alternans, where APs are out‐of‐phase across regions, exacerbate repolarization heterogeneity and is considered a potential trigger for VA development (Benoist et al., 2012). Mechanistically, this phenomenon has been linked to impaired Ca^2+^ handling and increased APD dispersion (Benoist et al., 2012). Notably, AP alternans is observed only in RV failure, but not in compensated RV, suggesting it represents a late stage change in electrical remodeling (Benoist et al., 2012). Its clinical significance in PH remains to be fully elucidated.

##### Calcium handling alterations

Beyond its effects on APD, alterations in calcium handling can result in delayed afterdepolarizations associated with increased diastolic Ca^2+^ leakage. Disruptions in calcium handling have been examined in detail in the MCT‐induced PH model. Tanaka et al. demonstrated that PH‐induced RV dysfunction is associated with increased Ca^2+^ spark frequency, prolonged Ca^2+^ transients, reduced sarcoplasmic reticulum Ca^2+^ content, and decreased calmodulin binding of ryanodine receptor 2 (RyR2). Stabilizing these receptors not only prevented Ca^2+^ leakage and structural remodeling but also reduced the incidence of VAs and mortality (Tanaka et al., 2022). Complementary findings by Wu et al. demonstrated altered expression of key calcium‐handling proteins, including phosphorylated calmodulin kinase II, sarcoplasmic/endoplasmic reticulum Ca^2+^‐ATPase (SERCA) 2a, the L‐Type calcium channel subunit alpha‐1C, along with elevated diastolic Ca^2+^ levels (the baseline intracellular calcium level during diastole), reduced Ca^2+^ transient amplitude, and diminished sarcoplasmic reticulum Ca^2+^ stores (Wu et al., 2022). These studies support the notion that improving calcium homeostasis may reduce vulnerability to VAs in PH (Wu et al., 2022) and underscore the central role of Ca^2+^ dysregulation in PH‐induced arrhythmogenesis (Tanaka et al., 2022; Wu et al., 2022).

### 3.2 Triggered mechanisms

#### 3.2.1 Autonomic dysregulation

PH is associated with significant ANS imbalance, characterized by heightened sympathetic activity and reduced parasympathetic tone (Ciarka et al., 2010; da Silva Goncalves Bos et al., 2018; Folino et al., 2003; Mak et al., 2012; Suzuki et al., 2012; Velez‐Roa et al., 2004; Wensel et al., 2009; Zimmer et al., 2020). This dysregulation correlates with both the hemodynamic subtype and World Health Organization Functional Classification of PH, as well as with the severity of concomitant RV dysfunction (Bienias et al., 2015; Zimmer et al., 2020). Preclinical studies have demonstrated that pharmacologic or neuromodulatory suppression of sympathetic activity attenuates PH progression and mitigates RV dysfunction (Ishikawa et al., 2009; Karpov et al., 2023; Na et al., 2014; Suzuki et al., 2012; Wang et al., 2024). In a chronic thromboembolic pulmonary hypertension rat model, an increased ratio of matrix metalloprotease‐2 and tissue inhibitor of metalloprotease‐1 in the lung as well as decreased fibroblast growth factor b levels in blood were proposed as a potential explanatory mechanism explaining beneficial effects of sympathetic denervation and parasympathetic stimulation on PH progression and RV function (Karpov et al., 2023). In MCT rat model, attenuation of sympathetic signaling was associated with increased nitric oxide bioavailability due to arginase inhibition (Na et al., 2014) and inflammatory immune responses (Wang et al., 2024) associated with improved vascular remodeling and RV function. Conversely, enhancement of parasympathetic activity improved survival, reduced pulmonary vascular remodeling, and preserved RV function in animal models (da Silva Goncalves Bos et al., 2018; Karpov et al., 2023; Yoshida et al., 2018, 2022).

Despite the link between ANS imbalance and RV remodeling, the role of ANS in ventricular arrhythmogenesis in PH remains poorly defined. Autonomic activity in PH has been assessed using heart rate variability parameters including the standard deviation of all normal‐to‐normal intervals (SDNN), the standard deviation of averages for all normal‐to‐normal intervals over 5‐min segments (SDANN), and the root mean square of successive differences (RMSSD) (Folino et al., 2003; Yi et al., 2014). SDNN and SDANN are reflective of sympathetic modulation, whereas RMSSD is considered a surrogate for parasympathetic activity (Folino et al., 2003; Yi et al., 2014). SDNN and SDANN are reduced in patients with idiopathic PAH compared with healthy controls, indicating sympathetic overactivity (Folino et al., 2003). Patients with more frequent VAs (PVCs >700/day) had a non‐significant trend toward lower SDNN and SDANN. Similarly, Yi et al. reported that patients with idiopathic PAH exhibited reduced heart rate variability indices and a higher frequency of premature ventricular ectopy compared to controls (Yi et al., 2014). Moreover, there was a significant inverse correlation between mPAP and heart rate variability parameters, reinforcing the association between hemodynamic severity, autonomic dysfunction, and ventricular ectopy (Yi et al., 2014).

However, the current evidence linking ANS dysregulation to VAs in PH is limited compared to other cardiovascular conditions such as heart failure or ischemic heart disease, highlighting the need for further mechanistic and clinical investigations to delineate the arrhythmogenic role of autonomic imbalance in the PH population.

#### 3.2.2 Ventricular ischemia

RV pressure overload in PH can precipitate myocardial ischemia. Evidence from myocardial scintigraphy, oxygen‐sensitive cardiovascular magnetic resonance (CMR) imaging, CMR flow quantification, and elevated circulating troponin levels supports the presence of ischemia in PH patients (Filusch et al., 2010; Gomez et al., 2001; Heresi et al., 2012; Sree Raman et al., 2021; Torbicki et al., 2003; van Wolferen et al., 2008). Ischemia alters both the ionic and metabolic milieu within the myocardium, promoting arrhythmogenesis. Ischemia results in extracellular acidosis, catecholamine release, and elevated potassium and lactate concentrations, and intracellular accumulation of Na^+^, Mg^2+^, Ca^2+^ with associated acidosis (Ghuran & Camm, 2001). These disruptions modify ionic fluxes across cardiomyocyte membranes, resulting in shortened APD, slowed CV, reduced upstroke velocity, and increased repolarization dispersion, thereby facilitating VAs through enhanced automaticity, triggered activity, and reentrant circuits, particularly in the setting of autonomic imbalance and fibrosis (Ghuran & Camm, 2001). Ischemic injury may also lead to cardiomyocyte death, indicated by troponin release, which correlates with adverse prognosis in PH (Filusch et al., 2010; Heresi et al., 2012; Torbicki et al., 2003). Thus, ischemia in the RV not only initiates arrhythmogenic mechanisms but also contributes to the chronic structural substrate sustaining VAs in the PH population.

#### 3.2.3 Inflammation and oxidative stress

Inflammation and oxidative stress are implicated in the pathogenesis of PH and RV remodeling (Al‐Qazazi et al., 2022; Brown et al., 2023; Hassoun et al., 2009; Rabinovitch et al., 2014; Rawat et al., 2014; Sun et al., 2017; Wang et al., 2017; Zimmer et al., 2020). These processes have also been linked to arrhythmogenesis in various cardiovascular conditions, including heart failure, myocardial infarction, and non‐ischemic cardiomyopathies (Table S2) (Adameova et al., 2020; Grune et al., 2021; Kouvas et al., 2018; Streitner et al., 2009; Yang et al., 2023).

RV overload, systemic inflammation, and myocardial ischemia in PH also provoke a localized inflammatory response within the RV. This immune activation initiates a chronic inflammatory cascade characterized by inflammatory cell recruitment and the upregulation of cytokines, chemokines, and adhesion molecules. Tumor necrosis factor‐alpha (TNF)‐α, interleukin‐6 (IL‐6), and interleukin‐1β (IL‐1β) are among the key mediators in this process (Dewachter & Dewachter, 2018), modulating ion channel expression and function, disrupting sarcoplasmic reticulum calcium cycling, and impairing gap junction integrity (Duncan et al., 2010; Grandy et al., 2010; Grune et al., 2021). TNF‐α downregulates Cx40 and Cx43, and alters their distribution, while also promoting apoptosis and fibroblast activation, creating a fibrotic substrate conducive to reentry (Haudek et al., 2007; Liew et al., 2013; Sawaya et al., 2007). IL‐6 suppresses the I_Kr_ current through activation of the soluble IL‐6 receptor (IL‐6R) and downstream Janus kinase signaling, thereby prolonging APD. Notably, this effect is reversible with IL‐6R blockade (Aromolaran et al., 2018). Similarly, IL‐1β inhibition improves CV and Ca^2+^ handling through upregulation of Cx43 and SERCA expression, while concomitantly reducing APD dispersion and VA susceptibility in animal models (De Jesus et al., 2017).

Additionally, emerging evidence highlights the electrophysiological influence of cardiac resident macrophages. These cells form gap junctions with cardiomyocytes via Cx43, modulating membrane potential and conduction (Fei et al., 2019; Hulsmans et al., 2017). Under physiological conditions, anti‐inflammatory cardiac resident macrophages (e.g., MHC‐II^low^CCR2^−^ and MHC‐II^high^CCR2^−^) support normal conduction, especially in the AV node, by promoting a more positive resting membrane potential and shortening APD (Epelman et al., 2014; Honold & Nahrendorf, 2018). However, inflammatory states promote recruitment of pro‐inflammatory monocyte‐derived macrophages (MHC‐II^high^CCR2^+^) to the myocardium, which prolongs APD, depolarizes the resting membrane potential, and increases myocyte capacitance via enhanced KCa3.1 channel activity and Ca^2+^ influx (Epelman et al., 2014; Fei et al., 2019; Honold & Nahrendorf, 2018). These monocyte‐derived macrophages also stimulate fibroblast activation and promote fibrosis, contributing to structural remodeling and reentrant arrhythmogenesis (Chen et al., 2020). In a Su/Pn rat model of PH, pronounced macrophage infiltration into the RV was correlated with increased RV fibrosis, hypertrophy, and impaired contractile function (Gu et al., 2022). These findings indicate that in the context of PH, macrophage‐mediated structural remodeling of the RV can synergize with electrophysiological alterations to enhance arrhythmia susceptibility.

In parallel, oxidative stress modulates electrophysiological properties by affecting ion channel function (Na^+^, K^+^, Ca^2+^), calcium homeostasis, and intracellular coupling via connexins (Adameova et al., 2020; Grandy et al., 2010; Grune et al., 2021). It enhances Kv11.1 (I_Kr_) current and suppresses voltage‐gated sodium channel alpha subunit 5 transcription, which encodes the cardiac Na^+^ channel (Adameova et al., 2020). Perturbations in Ca^2+^‐handling proteins including RyR2, SERCA, Ca^2+^/calmodulin‐dependent protein kinase II (CaMKII), and sodium‐calcium exchanger (NCX) are especially vulnerable to oxidative modifications. RyR2 oxidation increases sarcoplasmic reticulum Ca^2+^ leak, causing delayed afterdepolarizations, whereas SERCA oxidation impairs its activity and promotes cytosolic Ca^2+^ overload that prolongs APD. Oxidized CaMKII and NCX also further contribute to early and delayed afterdepolarizations, respectively (Adameova et al., 2020).

Collectively, ischemia, inflammation, and oxidative stress independently and synergistically drive the development of VAs in PH. These processes contribute to both electrophysiological remodeling through alterations in ion channel function, calcium dysregulation, and impaired cell–cell communication, and structural remodeling through fibrosis. Further elucidation of these mechanisms may facilitate the development of targeted therapeutic strategies to reduce arrhythmia susceptibility in patients with PH.

Despite these mechanistic insights, the available evidence is unevenly distributed between the two ventricles (Table S2). Most studies investigating oxidative stress and inflammation in pulmonary hypertension have focused on their contribution to RV remodeling, supporting their involvement in maladaptive hypertrophy, fibrosis, and progressive RV dysfunction. In contrast, the proarrhythmic effects of these pathways have been characterized predominantly in studies of the LV, where oxidative stress and inflammation have been linked to electrical remodeling, altered calcium handling, conduction abnormalities, and ventricular arrhythmogenesis. Consequently, direct evidence establishing a mechanistic link between oxidative stress, inflammation, and RV arrhythmogenesis remains scarce. This imbalance in the literature highlights an important knowledge gap and underscores the need for future mechanistic and translational studies to define how inflammatory and oxidative signaling contribute to RV electrical remodeling and arrhythmia susceptibility in PH.

## 4 TRANSLATIONAL AND THERAPEUTIC IMPLICATIONS

VAs in PH can result in a spectrum of clinical presentations, ranging from asymptomatic or minimally symptomatic episodes to sudden cardiac arrest in the general population (Al‐Khatib et al., 2018; Zeppenfeld et al., 2022). The clinical manifestations often depend on the type of arrhythmia and the degree of underlying structural remodeling. Given that PH is associated with significant RV hypertrophy, dilation, and overall cardiopulmonary dysfunction, affected patients may be particularly susceptible to the adverse effects of VAs.

Despite these pathophysiological vulnerabilities, current evidence suggests that PVCs and NSVT are generally not associated with significant symptoms, clinical deterioration, or adverse outcomes in PH populations (Andersen et al., 2021; Bandorski et al., 2015, 2023; Bandorski, Bogossian, Stempfl, et al., 2016). VF, although rare, has been documented as a cause of cardiopulmonary arrest in PAH patients (Hoeper et al., 2002). However, the clinical consequences and prognostic implications of sustained VT remain poorly defined due to the limited number of reported cases and the lack of systematic data.

These findings highlight the need for more robust clinical studies to determine which subtypes of VAs warrant intervention in PH and whether they serve as markers of advanced disease or direct contributors to mortality.

Currently, major clinical guidelines do not provide specific recommendations for the management of VAs in patients with PH (Al‐Khatib et al., 2018; Humbert et al., 2022; Zeppenfeld et al., 2022). Therefore, clinicians extrapolate from existing guidelines on ventricular arrhythmia management, which may be problematic given the unique pathophysiological mechanisms and clinical context of arrhythmias in PH.

### 4.1 Pharmacologic and procedural management of VAs

To date, no comparative studies have evaluated the efficacy and safety profiles of different classes of antiarrhythmic drugs specifically in patients with PH. Nonetheless, observational studies over the past two decades have reported the use of various agents including amiodarone, dronedarone, sotalol, flecainide, propafenone, beta‐blockers, and calcium channel antagonists, primarily in the treatment of supraventricular arrhythmias in this population (Olsson et al., 2013; Sammut et al., 2023; Tongers et al., 2007; Wen et al., 2014). The extrapolation of these findings to VA management remains uncertain and warrants further investigation.

Similarly, current guidelines do not provide specific recommendations for implantable cardioverter‐defibrillator use in patients with PH. It has been reported that complication rates among patients with PH undergoing device implantation may be higher, with lead dislodgement requiring repositioning occurring in up to 14% of cases (Reddy et al., 2012). While data on catheter ablation for VAs in PH are limited, a limited number of studies suggest that this may be a viable therapeutic option for PVCs and VT arising from the RV in PH patients (Bandorski et al., 2014) (Zou et al., 2021).

### 4.2 Mechanism‐based therapeutic targets emerging from experimental models

Recent preclinical studies have explored novel pharmacologic strategies aimed at mitigating VAs in PH by targeting RV structural and electrophysiological remodeling. Wu et al. and Qin et al. investigated the effects of dapagliflozin in the MCT model and demonstrated that the sodium‐glucose co‐transporter 2 inhibitor attenuated RV remodeling, improved calcium handling and electrophysiological parameters, and decreased susceptibility to VAs (Qin et al., 2022; Wu et al., 2022).

Similarly, Liles et al. reported that ranolazine, a late sodium current inhibitor, reduced electrical heterogeneity and improved RV structure and function, thereby preventing isoproterenol‐induced VT/VF in the MCT rat model (Liles et al., 2015). Tanaka et al. showed that dantrolene, a RyR2 stabilizer, completely suppressed epinephrine‐induced VT by inhibiting calcium sparks and spontaneous sarcoplasmic reticulum Ca^2+^ leakage (Tanaka et al., 2022). In addition, Cacheux et al. recently reported that extracellular cyclic adenosine monophosphate treatment reduced the incidence of VAs and suppressed pathological electrophysiological remodeling by reversing pathological cardiac and pulmonary vascular remodeling (Cacheux et al., 2025).

Collectively, these findings suggest that dapagliflozin, ranolazine, dantrolene, and extracellular cyclic adenosine monophosphate may reduce VA susceptibility in PH by modulating both structural and electrophysiological remodeling. However, these results remain preliminary, and further translational and clinical studies are warranted to evaluate their efficacy and safety in patients with PH.

## 5 KEY PHYSIOLOGICAL CONCEPTS

- Chronic RV pressure overload in PH promotes electrophysiological, structural, metabolic, inflammatory, and autonomic remodeling.
- RV action potential prolongation, repolarization dispersion, altered potassium and calcium currents, and calcium‐handling abnormalities may increase susceptibility to triggered activity and reentry.
- Fibrosis, cardiomyocyte hypertrophy, gap junction remodeling, and conduction heterogeneity create a structural substrate for ventricular arrhythmogenesis.
- Ischemia, inflammation, oxidative stress, and macrophage‐mediated electroimmune remodeling may amplify RV electrical instability.
- Most mechanistic evidence comes from preclinical models; integrative human physiological studies are needed.

## 6 LIMITATIONS OF EVIDENCE

Current evidence regarding ventricular arrhythmias in PH, particularly PAH, is limited by the lack of systematic ECG surveillance. Since prolonged rhythm monitoring is not routinely performed in clinical practice, the true incidence of VT and VF may be underestimated. In addition, the available studies are generally small and include relatively small numbers of patients with PAH. These limitations affect not only the accurate estimation of the epidemiology of VAs but also the assessment of their associated clinical manifestations and symptoms. Therefore, larger prospective studies with standardized rhythm monitoring are needed to accurately determine the prevalence and clinical significance of ventricular arrhythmias in this population, particularly among patients with advanced‐stage PAH.

Furthermore, mechanistic evidence linking oxidative stress and inflammation to ventricular arrhythmogenesis is largely derived from studies of the left ventricle, whereas direct evidence in the right ventricle remains scarce. Therefore, further mechanistic studies are needed to better define the relationship between oxidative stress, inflammation, and right ventricular arrhythmogenesis in PAH.

## 7 CONCLUSION

Ventricular arrhythmias are less common than supraventricular arrhythmias in patients with PH. The development of ventricular arrhythmias in pulmonary hypertension is likely multifactorial and reflects the combined effects of pressure overload‐induced RV remodeling, electrophysiological alterations, abnormal calcium handling, fibrosis, gap junction remodeling, autonomic imbalance, ischemia, inflammation, and oxidative stress. These changes may create both triggers and a substrate for ventricular arrhythmogenesis in the failing or maladapted right ventricle and may serve as therapeutic targets. The overall efficacy and safety profiles of traditional antiarrhythmic drugs, implantable cardioverter‐defibrillators, and catheter ablation have not been well studied among patients with VAs. Further investigation is needed to identify novel therapeutic approaches and establish evidence‐based strategies for risk stratification and treatment of VAs in this unique PH patient population.

## AUTHOR CONTRIBUTIONS

Study conception and design: Gaurav Choudhary, Abdulkadir Kiris. Drafting of manuscript: Gaurav Choudhary, Abdulkadir Kiris. Acquisition, analysis, or interpretation of data, and Critical review of manuscript: Abdulkadir Kiris, Peng Zhang, Peter Bronk, Bum‐Rak Choi, Gaurav Choudhary.

## CONFLICT OF INTEREST STATEMENT

The authors declare no conflicts of interest.

## ETHICS STATEMENT

The authors have nothing to report.

## Supporting information


**Table S1.** Search strategy.
**Table S2.** Effects of oxidative stress and inflammation on ventricular tissue.
