# Supplementary material for: Ventricular arrhythmogenesis in pulmonary hypertension: From right ventricular remodeling to clinical implications
Source: Physiol Rep. 2026 Aug 19;14(16):e71067. doi: 10.14814/phy2.71067 (PMC13490927; doi:10.14814/phy2.71067)
Supplement: Supplementary file 1 [file PHY2-14-e71067-s001.docx]

**SUPPLEMENTAL MATERIAL :**

**Literature search:**

This review was designed as a comprehensive narrative review. A comprehensive literature review search was conducted using PubMed, Scopus and Web of Science databases to identify studies published between 1960 and 2026. The search strategy employed the following keywords: pulmonary hypertension, pulmonary arterial hypertension, arrhythmia, ventricular arrhythmia, ventricular tachycardia, ventricular fibrillation, premature ventricular contractions, premature ventricular beats and ventricular ectopy (Supplementary Table 1). Boolean operators (AND/OR) were used to combine relevant search terms. The literature search and study selection were performed by two authors. Studies were selected based on the predefined eligibility criteria, including relevance to the association between VAs and PH, methodological quality, and clinical significance. Peer-reviewed original research articles and review articles offering insights into the association between VAs and PH were prioritized. Both human and experimental studies were considered when they provided relevant mechanistic or translational information; however, clinical studies were emphasized for epidemiological, prognostic, and therapeutic aspects. The final selection was guided by the quality of evidence, methodological rigor, and clinical relevance, with any uncertainties resolved by discussion among the authors. A total of 120 publications were included in the final review. This review is organized into sections addressing the epidemiology of VAs in PH, underlying pathophysiological mechanisms, translational and Therapeutic Implications, key physiological concepts and limitations of evidence.

| **Supplementary Table 1** | |
| --- | --- |
| **Database** | **Search strategy** |
| Pubmed | ("pulmonary hypertension" OR "pulmonary arterial hypertension")  AND  ("arrhythmia" OR  "ventricular arrhythmia" OR  "ventricular tachycardia" OR  "ventricular fibrillation" OR  "premature ventricular contractions" OR  "premature ventricular beats" OR  “ventricular ectopy”) |
| Scopus | Same search terms were used with database-specific syntax. |
| Web of Science | Same search terms were used with database-specific syntax. |

| **Supplementary Table 2. Effects of oxidative stress and inflammation on ventricular tissue** | | | | |
| --- | --- | --- | --- | --- |
| **Reference** | **Study population** | **Mechanisms** | **Effect** | **Relevant ventricle** |
| 71 | Rat | Oxidative stress, Inflammation | Remodeling | RV |
| 90 | Rat | Oxidative stress | Remodeling | RV |
| 91 | Rat | Oxidative stress | Remodeling | RV |
| 93 | Calve | Inflammation | Remodeling | RV |
| 94 | Rat | Inflammation | Remodeling | RV |
| 97 | Human | Inflammation | Arrhythmogenesis | LV |
| 98 | Human | Inflammation | Arrhythmogenesis | N/A |
| 101 | Rat | Inflammation | Arrhythmogenesis | N/A |
| 102 | Mice | Inflammation | Arrhythmogenesis | N/A |
| 105 | Mice | Inflammation | Remodeling | LV |
| 106 | Guinea pig | Inflammation | Arrhythmogenesis | N/A |
| 107 | Mice | Inflammation | Arrhythmogenesis | LV |
| 109 | Human | Inflammation | Arrhythmogenesis | LV |
| 113 | Rat | Inflammation | Remodeling | RV |
| LV: Left ventricle, N/A : not available, RV: Right ventricle | | | | |
